# Toward understanding the impacts of sediment contamination on a native fish species: transcriptional effects, EROD activity, and biliary PAH metabolites

**DOI:** 10.1186/s12302-016-0096-3

**Published:** 2016-12-05

**Authors:** Sven Koglin, Ulrike Kammann, Kathrin Eichbaum, Mathias Reininghaus, Bryanna Eisner, Steve Wiseman, Markus Hecker, Sebastian Buchinger, Georg Reifferscheid, Henner Hollert, Markus Brinkmann

**Affiliations:** 1 Department of Ecosystem Analysis, Institute for Environmental Research, RWTH Aachen University, Worringerweg 1, 52074 Aachen, Germany; 2 Institute for Pharmacy and Molecular Biotechnology (IPMB), Heidelberg University, Im Neuenheimer Feld 364, 69120 Heidelberg, Germany; 3 Thünen-Institute of Fisheries Ecology, Palmaille 9, 22767 Hamburg, Germany; 4 Toxicology Centre, University of Saskatchewan, 44 Campus Drive, Saskatoon, SK S7N 5B3 Canada; 5 Department of Biological Sciences, University of Lethbridge, 4401 University Drive, Lethbridge, AB T1K 3M4 Canada; 6 School of Environment and Sustainability, University of Saskatchewan, 44 Campus Drive, Saskatoon, SK S7N 5B3 Canada; 7 Department G3: Biochemistry, Ecotoxicology, Federal Institute of Hydrology (BFG), Am Mainzer Tor 1, 56068 Koblenz, Germany; 8 College of Resources and Environmental Science, Chongqing University, 1 Tiansheng Road Beibei, Chongqing, 400715 China; 9 College of Environmental Science and Engineering and State Key Laboratory of Pollution Control and Resource Reuse, Tongji University, 1239 Siping Road, Shanghai, China; 10 State Key Laboratory of Pollution Control and Resource Reuse, School of the Environment, Nanjing University, Nanjing, China

**Keywords:** Quantitative real-time RT-PCR, EROD, Non-model fish species, Real-time PCR array

## Abstract

**Background:**

Both frequency and intensity of flood events are expected to increase as a result of global climate change in the upcoming decades, potentially resulting in increased re-suspension of sediments in fluvial systems. Contamination of these re-suspended sediments with legacy contaminants, including dioxins and dioxin-like compounds (DLCs), as well as polycyclic aromatic hydrocarbons (PAHs) is of great ecotoxicological concern. DLCs, and to some extent also PAHs, exhibit their toxicity through activation of the aryl hydrocarbon receptor (AhR). However, interactions of DLCs with pathways other than those known to be mediated through the AhR are not fully understood to date.

**Methods:**

This study aimed to investigate molecular and biochemical effects in roach (*Rutilus rutilus)* during a 10 days exposure to suspensions of three natural sediments that differed in the level of DLC contamination. Concentrations of biliary PAH metabolites and hepatic 7-ethoxyresorufin-*O*-deethylase activity were quantified in exposed fish. Furthermore, the abundance of transcripts of several genes related to energy metabolism, response to oxidative stress, and apoptosis, as well as cytochrome P450 1A (*cyp1a*) was quantified.

**Results:**

Biliary PAH metabolites and activation of the AhR were confirmed as suitable early warning biomarkers of exposure to suspended sediments containing DLCs and PAHs that corresponded well with analytically determined concentrations of those contaminants. Although the abundances of transcripts of superoxide dismutase (*sod*), protein kinase c delta (*pkcd*), and ATP-binding cassette transporter c9 (*abcc9)* were altered by the treatment compared with unexposed control fish, none of these showed a time- or concentration-dependent response. The abundance of transcripts of pyruvate carboxylase (*pc*) and transferrin variant d (*tfd*) remained unaltered by the treatments.

**Conclusions:**

We have shown that contaminated sediments can become a risk for fish during re-suspension events (e.g., flooding and dredging). We have also demonstrated that roach, which are native to most European freshwater systems, are suitable sentinel species due to their great sensitivity and ecological relevance. Roach may be particularly suitable in future field studies to assess the toxicological concerns associated with the release of DLCs and PAHs during sediment re-suspension.

## Background

Frequency and intensity of extreme weather phenomena are expected to increase as a result of global climate change, leading to an anticipated increase of the occurrence of flood events in some geographic regions [30, 37, 63]. Disturbance and re-suspension of sediments during processes such as flood events and dredging activities can increase the bioavailability of adsorbed contaminants [26, 28, 40, 67]. Consequently, there is great concern about exposure of aquatic biota to sediment-bound contaminants resulting from the increased occurrence of flood events [72]. For example, Hollert et al. [28] found that toxic effects during flood events were caused by remobilization of particle-bound pollutants in the river Neckar, Germany. Furthermore, Wölz et al. [71] demonstrated that flood events with discharges corresponding to a 5-year average recurrence interval led to a tenfold increase in bioassay-derived toxicity equivalents of suspended particulate matter (SPM). Finally, several novel laboratory studies that combined methods from hydraulic engineering and ecotoxicology for exposure of rainbow trout (*Oncorhynchus mykiss*) to simulated flood events demonstrated that associated effects, including induction of biotransformation enzymes and genotoxicity, were elevated after exposure [8, 18, 29].

Of particular concern in this context are legacy contaminants including dioxins and dioxin-like compounds (DLCs), which are one of the most hazardous groups of chemicals currently known [5]. DLCs include planar halogenated hydrocarbons (PHHs), such as polychlorinated dibenzo-*p*-dioxins and furans (PCDD/Fs) and, polychlorinated biphenyls (PCBs). All DLCs share a typical planar structure, which leads to an elevated binding affinity to the aryl hydrocarbon receptor (AhR). Some polycyclic aromatic hydrocarbons (PAHs) also bind to the AhR and may induce dioxin-like effects. The chemical properties and (eco) toxicological effects of DLCs have been intensively studied in the past and remain the focus of many scientific investigations [68]. Measures have been taken to reduce the emission of DLCs into the environment (i.e., ban of PCBs, PCDD/F removal from flue gas); because of their hydrophobicity and persistence, however, DLCs strongly adsorb to sediments and have the potential to remain there for long periods of time [20, 36, 39, 48, 61, 69].

The effects of re-suspended sediments during flood events have been previously investigated in model species of fishes, such as the rainbow trout. However, less is known about effects of re-suspended sediments on non-model organisms such as the cyprinid *Rutilus rutilus* (roach). This species is eurytopic and omnivorous, and constitutes a significant proportion of the overall fish biomass in many European surface waters. Consequently, it has been proposed that roach could be a valuable model organism for ecological and ecotoxicological studies with a European focus [3, 42]. While there is a significant amount of knowledge regarding the basic biology and ecology of this species, less is known with respect to ecotoxicology. Studies have mostly focused on the effects of endocrine-disrupting chemicals in roach due to pollution of freshwater ecosystems by municipal and industrial wastewater effluents and water leakage from municipal landfills [6, 25, 32, 33, 41, 52, 58]. In other studies, the uptake of contaminants such as PCBs, PAHs, and heavy metals, and the induction of several biomarkers of exposure to these chemicals, including glutathione-*S*-transferase (GST) and 7-ethoxyresorufin-*O*-deethylase (EROD), have been investigated [16, 17, 34, 47, 65]. However, a very limited number of studies report the results of laboratory exposures of roach to single substances [11, 13, 14, 70].

Here, we investigated the consequences of exposure of roach to three different sediments characterized by differing levels of contamination with DLCs and PAHs. Sediments were collected from the rivers Rhine and Elbe. Contamination was lowest in Ehrenbreitstein (Rhine, Koblenz, Germany), greater in Prossen (Elbe, Germany), and greatest in Zollelbe (Elbe, Magdeburg, Germany) sediment. Analyses in exposed fish comprised of measures of the uptake of PAHs by means of high performance liquid chromatography analysis of bile liquid with fluorescence detection (HPLC-F). In addition, the effects of sediment exposure on the expression of a number of genes that were selected based on results of a previous study to characterize the effects of selected DLCs in roach by means of next generation high throughput sequencing of RNA (RNAseq) were investigated [11]. With this selection of genes, several important cellular pathways were surveyed: (a) phase I metabolism of xenobiotics and multidrug resistance (MDR) by quantifying expression of *cytochrome p450 1a* (*cyp1a*) [69] and the *ATP*-*binding cassette transporter c9* (*abcc9)* that encodes for the MDR- and potassium channel-regulating protein ABCC9 [15, 46]; (b) energy metabolism by quantifying expression of *pyruvate carboxylase* (*pc*) and *glycogen phosphorylase* (*pygl*), the products of which are important for synthesis of oxaloacetate for the citric acid cycle and gluconeogenesis (PC, [2, 31, 66] and liberation of glucose from glycogen, respectively (PYGL, [56, 57]; (c) apoptotic processes by measuring the expression of *protein kinase c delta* (*pkcd*) that regulates the expression and function of many apoptosis-related proteins [7, 12, 44, 49, 50]; (d) the response to oxidative stress by quantifying expression of *extracellular superoxide dismutase* (*sod*). SOD is one of several anti-oxidant enzymes that control concentrations of reactive oxygen species and catalyzes the dismutation of the superoxide radical (O_2_^−^) (reviewed by [22]; (e) the immune system by quantifying expression of *transferrin variant d* (*tfd*) which encodes an iron transporter that can deprive harmful microorganisms of this essential nutrient [51]. The main objectives of this study were to describe (1) uptake of DLCs and PAHs from re-suspended sediments, (2) the associated effects on molecular and biochemical responses in the roach, and (3) to verify the relevance of changes in the expression of the previously described genes under realistic exposure conditions.

## Methods

### Experimental design

In this study, roach were exposed to three samples of sediments contaminated with DLCs under simulated sediment re-suspension conditions. Effects of the three different contaminated sediments on gene expression were determined after 2, 6, and 10 days of exposure by comparing treatments to an unexposed control group. Abundances of transcripts of target genes were quantified by quantitative real-time reverse transcription PCR (qPCR). Furthermore, the qPCR results for *cyp1a* were complemented by determination of hepatic 7-ethoxyresorufin-*O*-deethylase (EROD) activity.

### Experimental animals—*Rutilus rutilus*

Juvenile roach were obtained from a local supplier (Inquadro, Aachen, Germany). Fish were allowed to acclimatize to laboratory conditions for at least 2 months prior to experimentation. Fish were held in 1000 L tanks that were aerated and supplied with dechlorinated municipal tap water (approx. 15 °C; pH 7.8 ± 0.2; NH_3_ < 0.1 mg L^−1^) by use of a flow-through system that exchanged water at a rate of 50–100% per day. Light and dark phases were 12 h each. Fish were fed ad libitum with frozen chironomids (Aquahobby, Peine, Germany). Fish were used in accordance with the Animal Welfare Act and with permission of the German federal authorities (registration number 84-02.04.2011.A368).

### Sediments used for exposure

Three riverine sediments (Zollelbe, Prossen, and Ehrenbreitstein) that had a similar distribution of particle size and physicochemical properties but differed in concentrations of DLCs and PAHs were used in exposures [23, 27]. The Zollelbe (ZE) sediment was sampled from a cut-off meander of the river Elbe in Magdeburg, Germany. The industrial history of this site as a shipping port resulted in sediments that are heavily contaminated with DLCs. The sampling location of the Prossen (PR) sediment was a small, non-industrial harbor at the river Elbe in Prossen, Germany, in close vicinity to the Czech border. Ehrenbreitstein (EBR) sediment was taken from a harbor located at the river Rhine in Koblenz, Germany. Total concentrations of PCDD/Fs, PCBs, the priority 16 PAHs of the United States Environmental Protection Agency (US-EPA), and the total organic carbon (TOC) content of each sediment were measured in the course of a previous study [10] and are provided in Table 1. All three sediments showed mainly silty and sandy characteristics. Furthermore, there was a gradient of the total concentrations of PCDD/Fs, PCBs, and PAHs in which ZE always showed the greatest, and EBR the lowest, contamination level.

**Table 1:** Sediment classification, location, as well as concentrations of PCDD/Fs, dl-PCBs, 16 EPA-PAHs, and total organic carbon in sediments

Sediment	Class	PCDD/F (ng WHO 2005 TEQ kg^−1^ dw)	PCB (ng WHO 2005 TEQ kg^−1^ dw)	Σ 16 EPA-PAH (mg kg^−1^ dw)	TOC (g kg^−1^ dw)
Zollelbe (+52° 7′ 47″ N, +11° 38′ 57″ E)	Silty sand	211.37	6.82	9.75	64.3
Prossen (+50° 55′ 40″ N, +14° 6′ 55″ E)	Sandy silt	10.25	2.85	6.45	63.1
Ehrenbreitstein (+50° 21′ 12″ N, +7° 36′ 27″ E)	Sandy silt	5.66	2.71	2.40	49.6

Fractionation and chemical analyses have been conducted by the German Federal Institute of Hydrology and were reported in Brinkmann et al. [10]

### Exposure of roach to sediments

Prior to initiation of exposures, sediments were homogenized using an electric stirrer and stored in 60 L aliquots at 4 °C. Experimental animals were exposed to the suspended sediments (1 g L^−1^) in glassfiber-reinforced plastic containers with a volume of 550 L (AGK Kronawitter, Wallersdorf, Germany). To maintain a constant concentration of particulate matter, water and sediment were constantly circulated within the containers by means of submersible pumps (maximum flow-through 6000 L h^−1^). A grid separated the containers into two parts to protect the fish from the pump. Each tank had a single source of aeration and the average water temperature was held constant at 17.5 ± 1.0 °C by use of stainless steel heat-exchangers. Temperature, pH, and concentration of dissolved oxygen (DO) were monitored daily using calibrated handheld instruments (Hanna, Ann Arbor, USA; WTW, Weilheim, Germany; VWR, Darmstadt, Germany). Water hardness and concentrations of suspended particulate matter (SPM) were measured every sampling day to ensure constant experimental conditions. For determination of concentrations of SPM, samples of suspended sediment were collected in 500 mL glass bottles and filtered through pre-weighed 0.7 μm glass fiber filters (MN-GF 1, Macherey & Nagel, Düren, Germany) using a Büchner funnel. Retained SPM and filters were dried at 105 °C overnight, weighed, and used to calculate concentrations of SPM. Total hardness was measured in the filtrate using the titrimetric Titriplex B method (Merck, Darmstadt, Germany). Measured concentrations of SPM ranged from 0.68 to 1.92 g L^−1^. Total hardness ranged from 58 to 78 mg L^−1^ CaO. Each exposure system was run with clean water for several days to reach stable water temperature and aeration of the water. The thoroughly mixed and weighed sediment was added to the water 1 day before starting the experiment so that it could distribute homogeneously.

Groups of 24 roach were carefully transferred into each container. For each treatment, one separate container was used. The fish (*n* = 6 per treatment) were sub-sampled at each time point. The animals were fed with frozen chironomids each day (10 g dw kg^−1^ fish day^−1^, Aquahobby) but food was withheld 2 days prior to sampling of fish. The length (mean ± standard deviation) of fish on day 0 of exposure was 116 ± 20 mm and the body mass (mean ± standard deviation) was 22.3 ± 13.0 g wet mass (ww), which corresponds to a loading rate of less than 1 g ww per liter of water (cf. OECD 203 [54], OECD 305 [55].

### Termination of exposure and organ preservation

Roach were sub-sampled at 0, 2, 6, and 10 days of exposure to sediments. Unexposed fish (day 0) served as control group. Fish were individually anesthetized with benzocaine (>250 mg L^−1^) and euthanized by exsanguination. The animals were opened ventrally, and bile liquid was sampled prior to removing the liver and placing it on ice. Next, 30 mg of liver tissue from each fish was transferred into RNAlater solution and samples were stored at −20 °C. In addition to the samples preserved in RNAlater, pieces of livers were snap-frozen in liquid nitrogen and stored at −80 °C for analysis of activities of 7-ethoxyresorufin-*O*-deethylase (EROD).

### Treatment of bile samples and HPLC analysis

Metabolites of PAHs in bile were quantified by use of a modification of the method published by Kammann et al. [35]. Because of limitations in sample quantity, samples from each time point per treatment were pooled (*n* = 1–3 per treatment and time point) for analysis. Briefly, 25 µL bile fluid was mixed with 95 µL distilled water and 5 µL *β*–glucuronidase/arylsulfatase solution (30/60 U mL^−1^) and subsequently incubated for 2 h at 37 °C. After stopping the reaction with 125 µL solution of 5 mg mL^−1^ ascorbic acid in ethanol, the mixture was centrifuged (700×*g*, 5 min) and the concentrations of 1-hydroxypyrene and 1-hydroxyphenanthrene were determined by means of HPLC with fluorescence detection [35]. The limits of detection (LODs) and quantification (LOQs) for 1-hydroxypyrene were 0.20 µg mL^−1^ (LOD) and 1.35 µg mL^−1^ (LOQ), and 0.03 µg mL^−1^ (LOD) and 0.10 µg mL^−1^ (LOQ) for 1-hydroxyphenanthrene, respectively.

### Quantitative real-time RT-PCR

Total RNA was extracted from individual samples of livers using the NucleoSpin RNA Mini kit (Macherey–Nagel, Düren, Germany) according to the manufacturer’s protocol, and RNA concentration was determined at 260 nm by use of a BioDrop *µ*LITE spectrophotometer (BioDrop, Cambridge, UK). RNA samples were stored at −80 °C until further processing. First-strand cDNA was synthesized from 1.5 μg of total RNA by use of the M-MuLV reverse transcriptase (Peqlab, Erlangen, Germany) according to manufacturer’s protocol. Primers for quantitative real-time PCR (qPCR) of selected transcripts (Table 2) were obtained from Eurofins Genomics (Ebersberg, Germany). The primers were designed using a database of the transcriptome of roach liver generated previously [11]. PCR products of cDNA samples were separated on 1% agarose gels along with a DNA size standard (New England Bioloabs, Frankfurt am Main, Germany) to confirm single PCR amplicons with the desired length. Melting curve analyses were performed during real-time PCR to ensure target specificity and single peak amplification, respectively. Furthermore, qPCR products were purified using the NucleoSpin Gel and PCR clean-up kit (Macherey–Nagel) according to the manufacturer’s protocol and the amplicon identity confirmed by DNA sequencing (Eurofins Genomics).

**Table 2:** Sequences of primer pairs and corresponding amplicon sizes used in quantitative real-time PCR [11]

Transcript	Primer sequence (5′–3′)	Amplicon size (bp)
Cytochrome P450 1a (*cyp1a*)	F: TTCGGAGCCGGTTTCGACAC R CCTCGAGGAGCGGCAGG	166
ATP-binding cassette transporter C9 (*abcc9*)	F: CAGGGATGCACACGACATC R ACGGGAAGGACGGAAGAATG	199
Pyruvate carboxylase (*pc*)	F: GTAAAGGTGAAGCCAGGCCAG R: TCCCCTTCCAGGCTGCTGTC	146
Glycogen phosphorylase liver isoform (*pygl*)	F: TGGCCAATCACAGGATCGTTA R: TTCTCAATTGCCTCCACGTCA	184
Protein kinase c delta type (*pkcd*)	F: TCCAGTAACAGCAACAGTTGAGA R: CCTGCTAGACATCTTGTTCATGC	200
Extracellular superoxide dismutase (*sod*)	F: GAGTTCGACAACACAATCTATGCCAC R: CAGCCTTGACTGAGGTCTCC	212
Transferrin variant d (*tfd*)	F: GGCACACTGGCAAGTTTACAT R: GGCTTTCAGGTGTCTTGCAG	198
*β*-Actin—reference gene (*actb*)	F: CCGTAAGGACTTGTATGCCAACAC R: GGTGGGGCAATGATCTTGATC	132

Abundance of mRNA was quantified by means of qPCR on a 96-well StepOne Plus real-time PCR system (Applied Biosystems, Foster City, CA, USA) using SYBR Green master mix (Applied Biosystems) as described in Brinkmann et al. [8]. Gene expression was normalized to the expression of the reference gene β-actin (*actb*) because abundance of transcripts of *actb* did not differ significantly between the control and treatment groups, indicating that this gene was suitable for normalization (One-way ANOVA, *p* = 0.49). Data was analyzed using the relative standard curve method as described in Rutledge and Cote [60]. The threshold was set to a ∆Rn = 0.4 and the baseline was determined from 3 to 15 cycles (software default value).

### EROD assay

The EROD assay was conducted as described by [29] and Brinkmann et al. [10]. For the measurement of fluorescence, a spectrofluorometer (Infinite M200, Tecan, Crailsheim, Germany; excitation: 530 nm, emission: 585 nm) was used. Three technical replicates of each sample were used. Concentrations of resorufin were interpolated from fluorescence values using linear regression. To calculate specific enzyme activities, protein concentrations were determined using the bicinchoninic acid (BCA) method with bovine serum albumin (BSA) as a standard (Sigma-Aldrich).

### Data analysis

All graphs and diagrams were created and statistically analyzed using GraphPad Prism 6.05 (GraphPad, San Diego, USA). Data identified as significant outliers (Grubb’s test) were omitted from further analyses. Fold changes of abundances of transcripts were *log*_*10*_-transformed prior to further statistical analysis since datasets did not pass Shapiro–Wilk’s normality test (*p* ≤ 0.05). Two-way ANOVA with Dunnett’s post hoc test (*p* ≤ 0.05) was used to identify significant differences between exposure groups.

## Results

### Biliary PAH metabolites

**Fig. 1:**
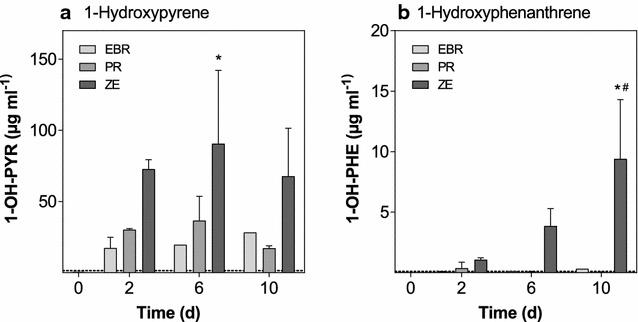
Biliary concentrations of 1-hydroxypyrene and 1-hydroxyphenathrene. Biliary concentrations of 1-hydroxypyrene (**a**, 1-OH-PYR) and 1-hydroxyphenathrene (**b**, 1-OH-PHE) were determined by means of HPLC with fluorescence detection. *Bars* represent the average of all pools measured for the respective treatment (*n* = 1–3) and the control (*white bars*),* error bars* the standard deviation. *Asterisks* indicate statistically significant effects compared to the control within each treatment, while pound signs indicate statistically significant differences compared to the EBR treatment within one time point (two-way ANOVA with Dunnett’s post hoc test, *p* ≤ 0.05)

Concentrations of 1-hydroxypyrene and 1-hydroxyphenanthrene in bile, both, showed an increase in concentrations with increasing exposure time (Fig. 1). In particular, for 1-hydroxypyrene, this increase was also concentration-dependent, and thus, reflected the differences in levels of contamination of the three sediments. The concentration of 1-hydroxypyrene in control fish was 0.45 µg mL^−1^ bile liquid and was significantly increased to 90.6 µg mL^−1^ after 6 days of exposure to ZE sediment. For 1-hydroxyphenanthrene, concentrations in bile from fish exposed to ZE sediment were significantly greater than concentrations in bile of fish exposed to the control and EBR sediment after 10 days of exposure.

### Abundance of *cyp1a* transcripts and EROD activity

**Fig. 2:**
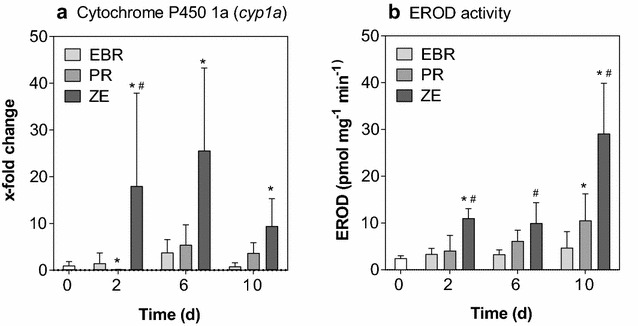
Abundance of *cyp1a* transcripts and EROD activity. Fold changes of the abundances of *cyp1a* transcripts as determined by means of quantitative real-time RT-PCR (**a**), as well as EROD activity induced after the realistic exposure scenario (**b**). *Bars* represent the average of all fish used for the respective treatment (*n* = 6) and the control (*white bars*), and *error bars* are the standard deviation. *Asterisks* indicate statistically significant effects compared to the control within each treatment, while pound signs indicate statistically significant differences compared to the EBR treatment within one time point (two-way ANOVA with Dunnett’s post hoc test, *p* ≤ 0.05; transcript abundance data was of *log*_10_-transformed)

Exposure to EBR sediment did not significantly alter the abundance of transcripts of *cyp1a* or EROD activity (Fig. 2). Compared to controls, abundance of transcripts of *cyp1a* was 18.0-, 25.6-, and 9.41-fold greater in livers from fish exposed to the ZE sediment and sampled at 2, 6, and 10 days, respectively. In comparison to fish exposed to the EBR sediment, the only significant difference in abundance of transcripts of *cyp1a* was in livers from fish sampled after 2 days of exposure to ZE sediment. In addition to the effects on *cyp1a* expression, EROD activity was significantly greater at 2 and 10 days in fish exposed to the ZE sediment, compared to activity in livers from control animals, and at all time points compared to livers from fish exposed to the EBR sediment. The highest induction of EROD activity was found after 10 days of exposure to the ZE sediment, where the activity rose to 29.1 pmol mg^−1^ min^−1^. In samples from fish exposed to the PR sediment, a significant 11.1-fold decrease in the abundance of transcripts of *cyp1a* compared to the control was observed after 2 days of exposure. No such significant changes were observed after 6 and 10 days of exposure to PR sediment. In contrast, exposure to PR sediment led to a significant increase in EROD activity only after 10 days compared to the control. As was observed in fish exposed to the ZE sediment, the highest induction of EROD activity was observed after 10 days of exposure to PR sediment.

### Abundance of transcripts of other studied genes

**Fig. 3:**
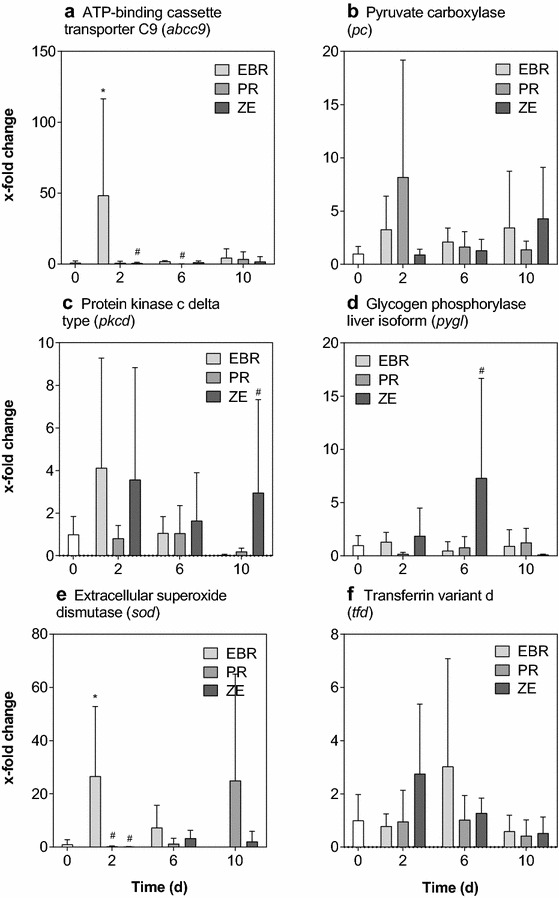
Fold changes of the abundances of six selected transcripts. Fold changes of the abundances of six selected transcripts as determined by means of quantitative real-time RT-PCR: ATP-binding cassette transporter c9 (*abcc9*, **a**), pyruvate carboxylase (*pc*, **b**), protein kinase c delta (*pkcd*, **c**), glycogen phosphorylase (*pygl*, **d**), superoxide dismutase (*sod*, **e**), and transferrin variant d (*tfd*, **f**). *Bars* represent the average of fish used for the respective treatment (*n* = 6) and the control (*white bars*), *error bars* the standard deviation. *Asterisks* indicate statistically significant effects compared to the control within each treatment, while pound signs indicate statistically significant differences compared to the EBR treatment within one time point (two-way ANOVA of *log*_10_-transformed data with Dunnett’s post hoc test, *p* ≤ 0.05)

In addition to *cyp1a*, effects of exposure to contaminated sediments on expression of six other genes (*abcc9, pc*, *pygl*, *pkcd*, *sod*, *tfd*) were quantified, for which no clear dose-dependent or temporal effects could be observed (Fig. 3). In livers from fish exposed to the EBR sediment for 2 days, abundance of transcripts of *abcc9* was 48.4-fold greater and the abundance of transcripts of *sod* was increased by 26.6-fold after 2 days of exposure to EBR sediment compared to the control. Expression of several genes of interest was significantly different in livers from fish exposed to the PR sediment and ZE sediment compared to fish exposed to the EBR sediment. Abundances of transcripts of *abcc9* at 6 days of exposure and *sod* at 2 days of exposure to the PR sediment were significantly different compared to fish exposed to the EBR sediment. Abundances of transcripts of *abcc9* and *sod* at 2 days, *pygl* at 6 days, and *pkcd* at 10 days were significantly different compared to fish exposed to EBR sediment. Abundances of transcripts of *pc* and *tfd* were not different in fish exposed to either sample of sediment.

## Discussion

In this study, exposure of roach to re-suspended samples of sediments from three locations along the Elbe and Rhine rivers contaminated with different concentrations of DLCs was assessed by quantifying concentrations of PAH metabolites in bile fluid, expression of *cyp1a* and EROD activity as biomarkers of activation of the AhR, and expression of several genes that were found to be dioxin-responsive in a previous study [11]. The surveyed genes encode for proteins that are part of important cellular processes, including biotransformation of xenobiotics, energy metabolism, and apoptosis.

### Uptake of PAHs from sediment suspensions

A number of studies have demonstrated that concentrations of PAH metabolites in bile from fish are correlated to contamination of surface waters and sediments by PAHs. Because it is one of the most frequently detected and most prominent biliary metabolites in fish, 1-hydroxypyrene and its conjugates are often used as surrogates to assess total PAH exposure [59]. In this study, concentrations of 1-hydroxypyrene in bile of fish exposed to sediments contaminated with DLCs increased with duration of exposure and clearly reflected differences in concentrations of PAHs in the three sediments. Because fish were fed uncontaminated feed, uptake of PAHs most likely occurred via the gill and, to a smaller proportion, also dermally [19, 38, 43, 73]. Results of this study confirm previously published research, in which similar findings were reported for a different fish species, namely rainbow trout (*Oncorhynchus mykiss*), exposed to sediment suspensions contaminated with PAHs [8–10]: The concentration of 1-hydroxypyrene in control fish was 0.45 µg mL^−1^ bile liquid in the present study, which was very similar to the concentration measured in unexposed rainbow trout (0.46 µg mL^−1^ bile liquid) as reported by Brinkmann et al. [10]. The maximum concentration of 1-hydroxypyrene in bile of roach exposed to ZE sediment was 90.6 µg mL^−1^ bile liquid, which is approx. 13-fold greater compared with the highest concentration previously measured in rainbow trout exposed to the same sediment sample (6.82 µg mL^−1^ bile liquid). This trend was even more pronounced for the concentrations of 1-hydroxyphenanthrene: while its maximum concentration in roach was as high as 9.39 µg mL^−1^ bile liquid, concentrations that were 35-fold lower (0.27 µg mL^−1^ bile liquid) were observed in rainbow trout [10]. The two studies differed mainly in the fact that exposure durations in the present study were much shorter (10 days) compared with the rainbow trout study (90 days). Therefore, it is likely that fish exposed to PAHs for extended durations may have more efficiently eliminated these chemicals [24]. Furthermore, inter-species differences in toxicokinetics between roach and rainbow trout might also influence concentrations of PAH metabolites in bile measured after exposure to contaminated sediment.

### AhR-mediated effects

Expression of *cyp1a* is directly regulated by the AhR signaling pathway. Activation of the AhR through binding of a ligand, i.e., DLCs, is required for its induction, which renders *cyp1a* expression and the related EROD activity one of the most widely used biomarkers of exposure to DLCs [69]. While the role of AhR signaling in adaptive responses to DLC exposure has been described in great detail, its contribution to the toxic effects of DLC is only poorly understood [45, 64].

The main objective of this study was to investigate the potential of *R. rutilus* to be used as a bioindicator for the detection of AhR activation following exposure to contaminated sediments under environmentally realistic exposure conditions. Abundance of transcripts of *cyp1a* was greater only in roach exposed to the ZE sediment. This is in contrast to results from another study with rainbow trout where each of the three sediments used in this study induced *cyp1a* mRNA levels after 7 days exposure [10]. In the same study, EROD activities were only significantly increased following prolonged exposure to ZE sediment, while in the present study, it was significantly elevated in roach exposed to ZE and PR sediment already after 2 and 6 days, respectively. Thus, it may be hypothesized that roach represent a more sensitive species compared to rainbow trout with regard to the induction of EROD activity. Abundance of transcripts of *cyp1a* and EROD activity was significantly greater after 2 days of exposure to ZE sediment and remained elevated for the duration of the exposure (Fig. 2a). Also, there was a statistically significant secondary increase in EROD activity after 10 days of exposure. Although abundances of transcripts of *cyp1a* were not significantly greater in livers from roach exposed to the PR sediment, EROD activity was increased. However, different from fish exposed to ZE sediment, EROD activity was not greater until 6 days of exposure.

Hepatic EROD activities and the abundance of transcripts of *cyp1a*, together with the previously discussed concentrations of biliary PAH metabolites, reflected the analytically determined concentrations of PCDD/Fs, PCBs, and PAHs in the sediments very well. The greatest concentrations of all investigated chemicals were found in ZE sediment: concentrations of PCDD/F were as high as 211.37 ng WHO 2005 TEQ kg^−1^ dw, concentrations of dl-PCBs were at 6.82 ng WHO 2005 TEQ kg^−1^ dw, and concentrations of the Σ 16 EPA-PAH were 9.75 mg kg^−1^ dw (Table 1). Concurrently, the greatest inductions of *cyp1a* transcript abundance, EROD activity, and biliary concentration of PAH metabolites were detected in the ZE treatments. The lowest concentrations of PCDD/Fs, PCBs, and PAHs of the present study were measured in EBR sediment, which also showed the least induction of *cyp1a* transcript abundance, EROD activity, and biliary PAH metabolites. Both in terms of concentrations of the measured DLCs and PAHs, as well as the observed biomarker responses, PR sediment was at an intermediate level. Thus, the investigated biomarkers can be considered sensitive and quantitative with regard to contamination with DLCs and PAHs.

Larmuseau et al. [42] previously acknowledged the suitability of roach as a model organism for ecology and fisheries research. In ecotoxicology, it has been shown that roach can be used as a bioindicator of endocrine disrupting chemicals [25, 32, 33, 52, 58] and DLCs in the environment, either by chemical analyses or quantification of biomarkers [16, 17, 47, 65]. In this study, we confirmed that activation of the AhR by DLCs and PAHs can also be detected in roach during environmentally relevant exposures to contaminated sediments.

### Effects on other dioxin-responsive genes under realistic exposure conditions

In addition to expression of *cyp1a*, abundances of transcripts of several other genes were quantified. These genes were identified previously, by use of RNAseq, as being differentially expressed in livers of roach injected with single DLCs, and were selected because they cover a number of important cellular functions. Inclusion of these additional genes, as done in the present study, was intended to provide information on other potentially relevant biological impacts of contaminated sediments. Assessing these genes in livers of roach exposed to the sediments included in this study also allowed for determination of whether they are suitable as biomarkers for use under field-like conditions.

Contrary to the results of our previous study, statistically significant changes in transcript abundance compared to unexposed control fish were only observed for *abcc9, pkcd,* and *sod*, and were neither strictly time- nor concentration-dependent. Interestingly, however, the abundance of transcripts of both *abcc9* and *sod* was significantly greater following exposure to EBR sediment for 2 days (Fig. 3), which may indicate an increased response to oxidative stress. In the study of Brinkmann et al. [11], a significantly lesser abundance of transcripts of *sod*, *pkcd*, *pygl,* and *tfd* was observed following injection of TCDD, while the abundance of transcripts of *abcc9* and *pc* was significantly greater. The results of these other genes investigated in the present study do not currently enable us to draw reliable conclusions on any effects on higher levels of biological organization linked to the respective genes. This may have several possible reasons: in the study of Brinkmann et al. [11], roach were injected with single DLCs, while in the present study, fish were exposed via the aqueous phase and DLCs were present in a complex mixture with other compounds that may also have an effect on the investigated genes. Furthermore, toxicokinetic differences between compounds will become more important when exposed via the aqueous phase instead of injection.

Findings of this study have important implications for the analysis of molecular markers in field-exposed and wild-caught animals: in the environment, chemicals are commonly present in complex mixtures, each constituent of which may (a) induce a response on its own contributing to the mixture effect in an additive manner, or (b) may show non-additive behavior by exhibiting antagonistic or synergistic effects [1, 21, 62]. Thus, if effects have been observed following exposure with single chemicals but not after exposure to sediments, the effect may have been modulated by other chemicals present in the sediments. Furthermore, conditions during exposure to sediment often comprise a number of other stressors, such as oxygen depletion and suspended particulate matter, which might also influence biomarker responses and, ultimately, the vulnerability of organisms to the effects of environmental contaminants [4, 53]. Therefore, we hypothesize that in the present study—apart from the fact that DLCs and PAHs were present in the investigated sediments—a combination of both the presence of other chemicals and the more complex exposure conditions may have led to a modulation of effects on the studied genes. In summary, none of the investigated candidate genes seemed to be sufficiently sensitive, responsive, and specific to exposure with DLCs and PAHs to be used as a biomarker of exposure to these substances.

## Conclusion

In the present study, we have shown that contaminated sediments can become a risk for aquatic biota, e.g., fish, during re-suspension (e.g., flood events, dredging). Biliary PAH metabolites and activation of the AhR, as assessed through hepatic abundance of transcripts of *cpy1a* and EROD activity, were confirmed as suitable early warning biomarkers of exposure to suspended sediments containing DLCs and PAHs that corresponded well with analytically determined concentrations of those contaminants. We have successfully demonstrated that native fish species, such as roach, are suitable sentinel species that can be used in future studies to assess the toxicological concerns associated with the release of DLCs and PAHs during sediment re-suspension in the field.
